# Performance of Ertapenem-Supplemented MacConkey Agar (MacErt) for Detecting Carbapenemase-Producing Enterobacterales

**DOI:** 10.7759/cureus.74106

**Published:** 2024-11-20

**Authors:** Ousmane Sow, Abdoulaye Cissé, Issa Ndiaye, Elhadj A Niang, Farma T Kane, Khoudia Cissé, Adja B Gueye, Aminatou A Bawa, Cheikh Fall, Yakhya Dieye, Bissoume Sambe, Abdoulaye Seck

**Affiliations:** 1 Microbiology, Institut Pasteur de Dakar, Dakar, SEN; 2 Medical Microbiology, Hôpital de Pikine, Dakar, SEN; 3 Epidemiology and Public Health, World Health Organization Regional Office for Africa (WCARO), Dakar, SEN; 4 Epidemiology and Public Health, Faculty of Medicine, Pharmacy, and Odonto-Stomatology, Université Cheikh Anta Diop de Dakar, Dakar, SEN

**Keywords:** amr surveillance, antimicrobial resistance (amr), carbapenemase-producing enterobacterales (cpes), carbapenem resistance, macconkey media supplemented with ertapenem, selective media, senegal

## Abstract

Background and objectives

Antimicrobial resistance (AMR) is a growing global threat, with carbapenemase-producing Enterobacterales (CPEs) representing a critical public health challenge. Rapid and accurate detection of CPEs is essential for controlling fatal bacterial AMR infections. This study evaluated the performance of MacConkey media supplemented with ertapenem (MacErt1 and MacErt2) for the detection of CPEs.

Methods

We formulated the media by supplementing MacConkey agar with ertapenem to final concentrations of 0.5 mg/L (MacErt1) and 1 mg/L (MacErt2). The media were assessed using a panel of 26 characterized Enterobacterales, including CPEs harboring oxacillinase (OXA)-48, OXA-181, New Delhi metallo-beta-lactamase (NDM)-5, and *Klebsiella pneumoniae* carbapenemase (KPC). All isolates were cultured on Mueller Hinton agar and incubated overnight at 36°C. Inocula were prepared and adjusted to a 0.5 McFarland standard. Ten microliter loops were used to streak MacErt1 and MacErt2 plates, which were then incubated overnight. After validation, MacErt1 was employed for the detection of CPEs in wastewater. A volume of 10 mL of wastewater was filtered, and the membrane was placed on MacErt agar, followed by overnight incubation. Grown colonies were identified using the Biotyper Sirius 2 MALDI-TOF (Bremen, Germany: Bruker), and the presence of carbapenem resistance genes was determined by lateral flow immunoassay (LFIA) tests and PCR.

Results

MacErt1 exhibited excellent sensitivity (100%) for all tested CPEs and a specificity of 77%. In contrast, MacErt2 demonstrated an overall sensitivity of 83%, primarily due to reduced sensitivity for OXA-181. However, it was 100% sensitive for detecting NDM, KPC, and OXA-48 producers. MacErt2 also maintained excellent specificity at 93%. The application of MacErt1 to wastewater samples resulted in 100% positivity and allowed the isolation of 124 CPEs among 150 examined isolates, predominantly NDM producers, followed by OXA-48-like and NDM+OXA-48-like strains. None of the isolates tested positive for blaKPC, blaVIM, or blaIMP.

Conclusion

This study demonstrated the efficacy of MacErt media for selectively detecting CPEs. MacErt1 exhibited 100% sensitivity for various CPEs and a specificity of 77%. MacErt2 showed 93% specificity and 100% sensitivity for NDM and KPC producers, making it suitable for targeted detection. These findings suggest that MacErt media provide an effective in-house solution for CPE surveillance, serving as a valuable tool in the ongoing battle against AMR.

## Introduction

Carbapenemase-producing Enterobacterales (CPEs) have emerged as a significant global concern, posing a major threat to public health, as carbapenems - once considered the last line of treatment for severe infections - are increasingly being compromised [1]. The capacity of CPEs to rapidly acquire additional resistance factors, coupled with their high potential for dissemination, underscores the critical importance of their detection and surveillance, particularly in settings with limited resources [2].

However, developing effective selective media for CPEs detection is challenging due to significant variations in carbapenemase enzyme hydrolysis [3,4]. Yet, important efforts have been made to advance carbapenem resistance monitoring, and several detection methods have been developed [5]. The direct MacConkey agar has been proposed for screening carbapenem-resistant Gram-negative rods in stool samples, but it demonstrated poor sensitivity [6]. Later on, the SUPERCARBA method showed promising results, achieving 96% sensitivity when compared to two commercial media, CHROMagar *Klebsiella pneumoniae* carbapenemase (KPC) and Brilliance CRE, but with relatively low specificity (68%) [7]. The use of cystine lactose electrolyte deficient (CLED) agar supplemented with multiple antibiotics, such as ceftazidime, cloxacillin, meropenem, and vancomycin, has also been tested [8]. The authors defined CRE as strains showing resistance to imipenem and/or meropenem (MIC ≥4 μg/mL) which may miss oxacillinase (OXA)-48-like producers that often remain susceptible to these carbapenems because of their weak hydrolytic activity. Commercial media, such as ChromID CARBA, have also shown limitations, particularly in detecting CREs with slightly reduced susceptibility to carbapenems, potentially underestimating the prevalence of CPEs [9].

Despite these challenges, it is worth noting that some selective methods have demonstrated good performance. MacConkey agar supplemented with meropenem (0.125 mg/L) and cefotaxime (1 mg/L) significantly improved sensitivity and specificity, ranging from 90.2% to 100% and 81.5% to 100%, respectively [9]. Similarly, the CHROMagar mSuperCARBA exhibited excellent diagnostic accuracy, with 93.05% sensitivity and 96.21% specificity, though it was more effective in detecting KPC and New Delhi metallo-beta-lactamase (NDM) carbapenemases as it failed to detect some OXA-48 producers [10].

In this study, we evaluated the performance of MacConkey agar supplemented with ertapenem (MacErt1 and MacErt2) for detecting CPEs. Our goal was to provide a cost-effective, non-commercial protocol that could be easily repeated, especially in resource-limited settings for CPEs detection and surveillance. Additionally, we conducted a pilot study aimed at assessing the utility of MacErt for CPEs surveillance in complex samples and providing an early picture of CPEs dissemination in wastewater systems in Dakar.

This article was previously published as a preprint on the Research Square server on January 29, 2024.

## Materials and methods

Bacterial isolates

The bacterial isolates were obtained from the Biobank of the Laboratoire de Biologie Médicale of Institut Pasteur de Dakar and comprised of 26 strains characterized by Enterobacterales strains, including *Escherichia coli*,* Klebsiella pneumoniae*,* Klebsiella oxytoca*,* Enterobacter cloacae*,* Citrobacter freundii*,and* Morganella morganii*, carrying various resistance determinants. Among these, 13 were carbapenem-resistant Enterobacterales (CREs) defined by ertapenem MIC ≥0.5 mg/L. Twelve of the 13 CREs were CPEs, harboring NDM-5 (n=5), OXA-181 (n=4), OXA-48 (n=1), KPC-3 (n=1), or a combination of NDM-5 and OXA-48 (n=1). The remaining CRE isolate was identified as an extended spectrum beta-lactamase (ESBL)-positive strain with a porin mutation. The other 13 isolates (13/26) were carbapenem-susceptible with ertapenem MIC ≤0.5 mg/L used as negative controls (Table 1).

**Table 1:** Bacterial isolates and MacErt media characteristics. AST: antimicrobial susceptibility testing; ESBLs: extended spectrum beta-lactamases; ETP: ertapenem; IMP: imipenem; MIC: minimal inhibitory concentration; CRGs: carbapenem resistance gene; S: susceptible; R: resistant; +: presence; -: absence; NDM: New Delhi metallo-beta-lactamase; KPC: *Klebsiella pneumoniae* carbapenemase; OXA: oxacillinase

ID	Species	AST			Minimal inhibitory concentrations					Bacterial counts (CFUs)		
		ESBL	ETP		ETP		IPM		CRGs			
			Diameters (mm)	Interpretation	MIC (µg/mL)	Interpretation	MIC (µg/mL)	Interpretation		MacConkey	MacErt1	MacErt2
ACTC1241	E. coli	-		S	≤0.5	S	≤0.25	S	-	≥200	0	0
ACTC15810	E. coli	-	35	S	≤0.5	S	≤0.25	S	-	≥200	0	0
Yd11	E. coli	-	35	S	≤0.5	S	≤0.25	S	-	≥200	0	0
Yd27	K. pneumoniae	+	27	S	≤0.5	S	≤0.25	S	-	≥200	0	0
Yd31	K. oxytoca	-	29	S	≤0.5	S	≤0.25	S	-	≥200	0	0
Yd37	E. coli	+	20	R	≤0.5	R	1	S	-	≥200	18	0
Yd43	E. cloacae	-	26	S	≤0.5	S	≤0.25	S	-	≥200	0	0
Yd45	E. coli	+	25	S	≤0.5	S	≤0.25	S	-	≥200	0	0
Yd46	E. coli	+	31	S	≤0.5	S	≤0.25	S	-	≥200	0	0
Yd49	E. coli	+	20	R	≤0.5	S	≤0.25	S	-	≥200	≥200	3
YD52	E. coli	+	29	S	≤0.5	S	≤0.25	S	-	≥200	0	0
Yd58	E. coli	+	24	R	≤0.5	S	≤0.25	S	-	≥200	12	0
Yd65	E. coli	-	17	R	≤0.5	S	≤0.25	S	-	≥200	0	0
Yd68	K. pneumoniae	+	32	S	≤0.5	S	≤0.25	S	-	≥200	0	0
BAA2814	K. pneumoniae	-		R	≥8	R	≥16	R	KPC-3	≥200	≥200	≥200
Yd2	E. coli	-	6	R	≥8	R	≥16	R	NDM-5	≥200	≥200	≥200
Yd22	M. morgannnii	-	6	R	≥8	R	8	R	NDM-5	≥200	≥200	≥200
Yd32	K. pneumoniae	-	6	R	≥8	R	8	R	NDM-5	≥200	≥200	≥200
Yd50	E. coli	+	6	R	≥8	R	8	R	NDM-5	≥200	≥200	≥200
YD62	K. pneumoniae	-	9	R	≥8	R	≥16	R	NDM-5	≥200	≥200	≥200
Yd59	C. freundii	+	6	R	≥8	R	≥16	R	NDM-5+OXA-48	≥200	≥200	≥200
Yd1	E. cloacae	-	10	R	≥8	R	2	S	OXA-48	≥200	≥200	≥200
Yd3	K. pneumoniae	-	17	R	2	R	2	S	OXA-181	≥200	≥200	0
Yd9	E. coli	-	19	R	2	R	≤0.25	S	OXA-181	≥200	≥200	0
Yd60	E. coli	-	19	R	4	R	≤0.25	S	OXA-181	≥200	≥200	5
Yd67	E. coli	-	17	R	4	R	≤0.25	S	OXA-181	≥200	≥200	7

MacErt media preparation

To prepare MacErt agar, 47.4 g of MacConkey powder (Marne-la-Coquette, France: Bio-Rad) was dissolved in distilled water, typically at a concentration of 47.4 g/L. The mixture was heated while stirring until the agar was completely dissolved. Subsequently, the solution was sterilized by autoclaving at 121°C for 15 min. Following sterilization, the agar solution was cooled to 40°-50°C. Separately, 10 mg of ertapenem (St. Louis, MO: Sigma-Aldrich) was dissolved in 20 mL of ultrapure water to achieve a final concentration of 0.5 mg/mL and stored at -20°C. Using aseptic techniques, 1 mL of the ertapenem solution was added to 1 L of cooled MacConkey agar to prepare MacErt1, while 2 mL of the solution was added to 1 L of cooled MacConkey agar for MacErt2. After adding the antibiotic, the media were gently mixed by inverting the containers several times. The agar-ertapenem mixture was then poured into sterile petri dishes (Durham, NC: Corning Gosselin) and allowed to solidify.

MacErt1 and MacErt2 evaluation

To assess the sensitivity and specificity of the media, 26 isolates including CPEs and negative controls were prepared. All the isolates were cultured on Mueller Hinton agar (Marne-la-Coquette, France: Bio-Rad) and incubated at 36°C overnight. Inocula were then prepared from these overnight cultures in saline water and adjusted to a 0.5 McFarland standard using a densitometer (Marcy-l'Étoile, France: BioMérieux). Ten microliter loops (Radnor, PA: VWR International) were used to streak the MacErt1 and MacErt2 plates. The plates were then incubated overnight, and the colony counts for each MacErt medium were estimated.

Application of MacErt1 to wastewater samples

Thirty wastewater samples were collected from community sewage, hospital wastewater, and at different points of the wastewater treatment plant of Hann, Dakar. At the sampling sites, a sump was used to collect water into 500 mL bottles. The samples were quickly transported to the laboratory in ice boxes. The physical and chemical parameters of the samples were directly determined onsite, and recorded in comprehensive form with Epicollect5 (https://five.epicollect.net/) (University of Oxford: Oxford, England). Other important data, such as location, water treatment, and the potential risk of direct contamination to humans and/or animals, were also recorded using the Epicollect5.

For each sample, 10 mL was filtered through a 47 mm sterile membrane with a 0.45 µm pore size (Molsheim, France: Millipore). The membrane was then placed on MacErt1 plates and incubated at 36°C overnight. After incubation, five well-isolated colonies were randomly selected and identified using the Biotyper Sirius 2 MALDI-TOF (Bremen, Germany: Bruker). Enterobacterales colonies were subsequently further characterized to assess the presence of carbapenemases.

Carbapenemase typing

All Enterobacterales isolates were subjected to a comprehensive analysis to identify carbapenem resistance determinants. Initially, the Enterobacterales isolates were subjected to lateral flow immunoassays (LFIA) tests, to determine the presence of carbapenemases using NG-Test CARBA-5 following the manufacturer's instructions (Guipry, France: NG Biotech). Subsequently, classical PCR was employed to confirm the presence of these resistance genes. Briefly, total DNA was extracted using the thermal lysis method, and PCR reactions were carried out with specific primers targeting bla_OXA-48_-like, bla_NDM,_ bla_KPC_, bla_VIM_, and bla_IMP_ genes, as previously described [11]. Positive, negative, and non-template controls were included in the PCR reactions. The amplification steps and the number of cycles were identical for all the genes, except for bla_IMP_ (Table 2).

**Table 2:** Polymerase chain reaction program. iDenat: initial denaturation; Denat.: denaturation; fExtension: final extension.

Targets genes (height)	iDenat.	Denat.	Annealing	Extension	fExtension	Cycles
bla_IMP_ (188 pb)	94°C, 4 min	94°C, 40 s	52°C, 40 s	72°C, 45 s	72°C, 4 min	n=35
bla_NDM_ (621 pb)	95°C, 3 min	94°C, 30 s	58°C, 1 min	72°C, 1 min	72°C, 7 min	n=30
bla_OXA-48_-like (743 pb)	95°C, 3 min	94°C, 30 s	58°C, 1 min	72°C, 1 min	72°C, 7 min	n=30
bla_VIM2004_ (382 pb)	95°C, 3 min	94°C, 30 s	58°C, 1 min	72°C, 1 min	72°C, 7 min	n=30
bla_KPC_ (795 pb)	95°C, 3 min	94°C, 30 s	58°C, 1 min	72°C, 1 min	72°C, 7 min	n=30

Following PCR amplification, 12.5 µL of each amplicon was separated on a 1.5% agarose gel in 1× TAE buffer for 30 min at 135 volts. The amplified DNA fragments were visualized using a GelDoc imager (Marne-la-Coquette, France: Bio-Rad).

Data collection and analysis

The data were collected and managed using Microsoft Excel (2016) (Redmond, WA: Microsoft Corp.), with the figures generated through this software. Statistical analyses including sensitivity and specificity of the media were conducted with R version 4.2.3 (Vienna, Austria: R Core Team).

## Results

Performances of MacErt1 and MacErt2

MacErt1 demonstrated excellent sensitivity (100%) across all CPEs, with a specificity of 77%. The reduced specificity was attributed to false positives, as indicated by the growth of three extended-spectrum beta-lactamase (ESBL)-producing *E. coli* strains. MacErt2 exhibited a sensitivity of 83% and a specificity of 93%, successfully recovering all CPEs, including NDM, KPC, OXA-48, and OXA-181 producers. However, two OXA-181-producing isolates with a MIC ≤2 µg/mL failed to grow on MacErt2.

MacErt1 showed lower specificity (77%) compared to MacErt2 (93%). Three false positives were noted with MacErt1 and one with MacErt2, all of which were ESBL-producing *E. coli *strains with reduced susceptibility to ertapenem (inhibition zone diameters of around 20 mm). Despite their growth, the colony counts were small. On MacErt1, colony counts were 12, 18, and ≥200 CFUs, respectively. Only the last strain (≥200 CFUs on MacErt1) managed to grow on MacErt2 but with a very low colony count of 3 CFUs (Table 1).

Wastewater analysis results

**Figure 1:**
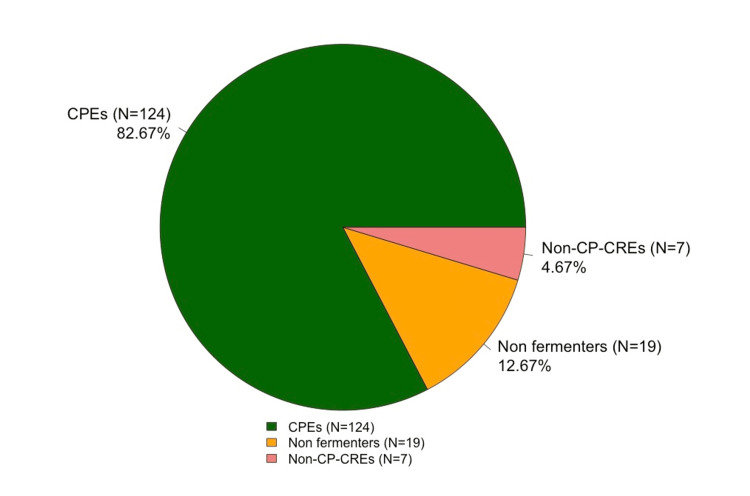
Distribution of CPE and non-CPE isolates identified in wastewater samples with MacErt1. CPEs: carbapenemase-producing Enterobacterales; non-CP CREs: non-carbapenemase producing carbapenem-resistant Enterobacterales Non-enterobacterales are bacterial isolates that do not belong to the Enterobacterales order.

All samples tested positive for at least one CPE. Among the 150 examined isolates, 82.67% (124/150) were CPEs, 4.67% (7/150) were non-carbapenemase-producing CREs, and 12.67% (19/150) were non-Enterobacterales Gram-negative bacilli, mostly Acinetobacter species (Figure 1).

The detected CPEs (n=124) were predominantly composed of *E. coli *(60%) and *K. pneumoniae* (33%). *Enterobacter cloacae, Citrobacter freundii, Citrobacter braakii, *and* Raoultella terrigena *all together accounted for 7%. The PCR and LFIA tests were concordant and showed the presence of NDM (65%) and OXA-48-like (31%). Notably, a co-occurrence of NDM and OXA-48-like was found in 4% of the CPEs. Another valuable epidemiologic information is that none of the isolates tested positive for blaKPC, blaVIM, or blaIMP.

## Discussion

The dissemination of CPEs remains a significant challenge in clinical settings. However, many laboratories in resource-constrained regions, in particular, are unable to effectively monitor these pathogens due to limited screening means. To address this issue, we evaluated MacConkey agar supplemented with ertapenem and applied it to wastewater samples. The objective of this study was to provide a simple and selective method for CPEs monitoring and evaluate whether the media could be a viable tool for routine surveillance. We used NDM, KPC, OXA-48, and OXA-181-producing isolates, including negative controls with known resistance profiles. Then, we applied the method in real-world conditions to conduct a pilot study and gain early insights into the environmental dissemination of CPEs through wastewater in Dakar, Senegal.

MacErt1 demonstrated 100% sensitivity across all tested CPEs, with a specificity of 77%. This high sensitivity is a significant advantage, particularly in epidemiological studies, as it successfully detected all carbapenemase types, including those with low ertapenem minimum inhibitory concentrations (MICs). Its reduced specificity was attributable to false positives, primarily from ESBL-producing *E. coli*, likely due to porin mutations that reduce carbapenem susceptibility. In fact, the combination of ESBL production with porin mutations is known to cause carbapenem resistance [12,13]. Similar mechanisms reduced the specificity of CHROMagar mSuperCARBA, one of the most accurate methods to date [10].

MacErt2, on the other hand, showed a sensitivity of 83% across all types of CPEs, with a specificity of 93%. Notably, MacErt2 successfully recovered all NDM, KPC, and OXA-48 producers, along with some OXA-181 producers. However, OXA-181 producers with MICs ≤2 µg/mL did not grow on MacErt2, which limits its sensitivity for this specific subgroup. Despite this, MacErt2's higher specificity and its ability to detect the clinically relevant carbapenemases - NDM, KPC, and OXA-48 - make it a strong candidate for clinical application in regions where these carbapenemase producers dominate.

Both MacErt1 and MacErt2 offer distinct advantages depending on the context of use. MacErt1, with its excellent sensitivity across all CPE types, is highly suitable for epidemiological studies where a broad detection spectrum is required. However, its lower specificity, suggests that confirmatory testing may be necessary to avoid overestimating CPE prevalence. On the other hand, MacErt2, with its higher specificity, offers a more targeted approach and is particularly valuable in clinical settings where rapid and accurate detection is crucial. Its slight limitation in detecting OXA-181 producers with lower ertapenem MICs may be less clinically significant in regions where NDM, KPC, and OXA-48 are predominant carbapenemases.

Previously, some agar supplemented with antibiotics approaches have been proposed for the isolation of CPEs. The MacConkey agar supplemented with 1 µg of imipenem reported a sensitivity of 84.9% and a specificity of 94.3% [14]. However, this study was limited by the small sample size (n=10), and the isolates were predominantly KPC-producers (KPC=8/10, VIM=IMP=1/10), which may not necessarily reflect sensitivity for the other carbapenemases. The development of SUPERCARBA media, later commercialized as CHROMagar SuperCARBA, marked a significant advancement in CPE detection, demonstrating sensitivities as high as 97% and a specificity of 88.2% [15]. While these findings are comparable to ours, our method offers distinct advantages. A key advantage of our MacErt1 medium is its low cost of €0.65 per plate. Additionally, MacErt is not intended for commercial use; rather, it serves as an alternative solution for microbiology labs in low- and middle-income countries (LMICs), where shipping laboratory consumables from the West can take months, hindering antimicrobial resistance (AMR) surveillance. MacErt can be easily prepared in-house with readily available components, and requires minimal training, making it accessible even to microbiologists with limited laboratory experience.

The wastewater surveillance pilot study provided key insights into the efficacy of MacErt1 in detecting viable CPEs in complex matrices like wastewater. The study identified CPEs in all tested samples, revealing the dominance of NDM and OXA-48-like carbapenemase types, with no isolates producing KPC*, *VIM, or IMP. These findings underscore the predominance of NDM and OXA-48-like enzymes in the Senegalese environment. This corroborates the tendency in clinical settings in Senegal [16]. KPC, VIM, or IMP have not been yet described in Senegal in contrast to OXA-48-like and NDM [16,17]. A study spanning two-year surveillance of CPEs in the clinical sector reported OXA-48-like, NDM-5, and the absence of KPC, VIM, and IMP (unpublished data). NDM and OXA-48-like have been previously reported in many African countries including Senegal, even if their magnitude is difficult to estimate due to data scarcity [18]. In contrast, blaKPC and blaVIM have been mostly reported in the United States and Europe and Asia, respectively [19,20]. The unsuspicious discovery of CPEs co-harboring NDM and OXA-48-like is a significant step forward in the scalation of AMR burden in Senegal. This highlights the growing burden of antimicrobial resistance (AMR) and underscores the potential value of MacErt media for early detection of CPEs.

Limitations

Despite the importance of our findings, a few limitations should be acknowledged in our study. Firstly, the assessment was conducted using a panel of 26 isolates, which may restrict the robustness of statistical analysis. Secondly, while the isolates used for validation were clinical, the media were not tested with clinical samples. Lastly, the geographical focus of the study may limit the generalizability of our results to other regions with different environmental conditions and bacterial populations.

## Conclusions

Overall, our study demonstrated the effectiveness of MacErt1 and MacErt2 as practical, low-cost alternatives for detecting carbapenemase-producing Enterobacterales (CPEs) in resource-limited settings. MacErt1 achieved 100% sensitivity and 77% specificity, successfully detecting circulating CPEs in the environment, primarily OXA-48-like and NDM producers, reported for the first time in Senegal. MacErt2, with 93% specificity and 83% sensitivity, proved highly effective for detecting NDM and KPC producers, making it a valuable tool in regions where these carbapenemases are predominant. Further research should investigate the genomic features of these environmental CPEs.
